# Urine Output During Cardiopulmonary Bypass Predicts Acute Kidney Injury After Cardiac Surgery: A Single-Center Retrospective Analysis

**DOI:** 10.1097/MD.0000000000003757

**Published:** 2016-06-03

**Authors:** Young Song, Dong Wook Kim, Young Lan Kwak, Beom Seok Kim, Hyung Min Joo, Jin Woo Ju, Young Chul Yoo

**Affiliations:** From the Department of Anesthesiology and Pain Medicine (YS, YLK, HMJ, JWJ, YCY); Anesthesia and Pain Research Institute (YS, YLK, YCY), Yonsei University College of Medicine, Seoul; Department of Policy Research Affairs (DWK), National Health Insurance Corporation Ilsan Hospital, Goyang; and Division of Nephrology (BSK), Department of Internal Medicine, Yonsei University College of Medicine, Seoul, South Korea.

## Abstract

Urine output is closely associated with renal function and has been used as a diagnostic criterion for acute kidney injury (AKI). However, urine output during cardiopulmonary bypass (CPB) has never been identified as a predictor of postoperative AKI. Considering altered renal homeostasis during CPB, we made a comprehensible approach to CPB urine output and evaluated its predictability for AKI.

Patients undergoing cardiovascular surgery with the use of CPB, between January 2009 and December 2011, were retrospectively reviewed. AKI was defined as an increase in serum creatinine ≥0.3 mg/dL in the first postoperative 48 hours. We extrapolated a possible optimal amount of urine output from the plot of probability of AKI development according to CPB urine output. After separating patients by the predicted optimal value, we performed stepwise logistic regression analyses to find potential predictors of AKI in both subgroups.

A total of 696 patients were analyzed. The amount of CPB urine output had a biphasic association with the incidence of AKI using 4 mL/kg/h as a boundary value. In a multivariate logistic regression to find predictors for AKI in entire patients, CPB urine output did not show statistical significance. After separating patients into subgroups with CPB urine output below and over 4 mL/kg/h, it was identified as an independent predictor for AKI with the odds ratio of 0.43 (confidence interval 0.30–0.61) and 1.11 (confidence interval 1.02–1.20), respectively.

The amount of urine output during CPB with careful analysis may serve as a simple and feasible method to predict the development of AKI after cardiac surgery at an early time point.

## INTRODUCTION

Acute kidney injury (AKI) is one of the most serious and potentially life-threatening complications after cardiac surgery.^1–3^ Because an early detection of AKI makes treatment quick and mitigates the progression of renal injury, efforts for early detection of patients at risk of AKI have been made using several risk stratification models.^4–6^

Among the diagnostic parameters, urine output is the only available bedside test for a kidney function. Oliguria is a major diagnostic criterion of AKI^7^ and is often used as a real-time indicator of AKI in critically ill patients.^8^ Moreover, its superiority over the serum creatinine in the early diagnosis of AKI has been suggested.^9,10^ Recently, it was reported that intraoperative oliguria was an independent risk factor for predicting AKI after aortic surgery.^11^ However, urine output during cardiopulmonary bypass (CPB) has never been identified as a predictor of AKI in a large number of risk models announced to date.^12–14^ Moreover, there is no consensus on the optimal amount of urine output during CPB.

As impairment of tubular reabsorption and heterogeneity of nephron function could paradoxically increase amount of urine output,^15^ the maintenance of urine flow may not guarantee a normally functioning kidney. In the same context, a large amount of urine output during CPB should not be interpreted as a favorable sign, because the tubular damage triggered by inflammatory and thrombotic response during CPB may increase the urine flow.^16^

We hypothesized that a relationship between the amount of urine output during CPB and the development of postoperative AKI may not be linear, but it is rather U-shaped. Moreover, we conducted a hypothesis-generating analysis to investigate the possible independent association between them.

## METHODS

### Study Population and Data Collection

After approval of Institutional Review Board, we retrospectively reviewed prospectively entered, protocol-based electronic medical records of all adult patients who underwent cardiovascular surgery with CPB at the Cardiovascular Hospital of Yonsei University Health System between January 2009 and December 2011 (n = 727). The need to obtain written consent from patients was waived. Patients who had had preoperative renal failure requiring dialysis (n = 21) and those without known preoperative serum creatinine levels (n = 10) were excluded. After careful examination of data, 696 patients were enrolled in the current study.

Standardized general anesthesia was provided to all patients. CPB was facilitated by a roller pump using a circuit primed with 1600 mL of solution comprising 6% hydroxyethyl starch 130/0.4, 20% mannitol (5 mL/kg), NaHCO_3_ (40 mEq), and acetated Ringer solution. Pump flows of 2.2 to 2.5 L/min/m^2^ and mean arterial pressure ≥60 mm Hg were targeted during CPB. Hemofiltration was performed during CPB in all patients having sufficient intravascular volume. After surgery, patients were transferred to the intensive care unit (ICU) and provided standardized postoperative care.

### Assessed Parameters

Preoperative data used in our analysis were age, sex, height, weight, New York Heart Association class, history of diabetes, hypertension, and cerebral vascular accident, additive EuroSCORE, and estimated glomerular filtration rate (eGFR) derived from serum creatinine 1 to 2 days before the surgery, which was calculated using the Chronic Kidney Disease Epidemiology Collaboration (CKD-EPI) Eq.^17^

Operative features including type of surgery, CPB and aortic cross clamp time, use of total circulatory arrest, fluid input and transfusion of blood products, urine output during CPB and total operation time, volume of hemofiltration, inotropes and vasoconstrictors administered, and use of diuretics were included in the current analysis.

As for the postoperative data, fluid input, transfusion of blood products, urine output, inotropes and vasoconstrictors administered during 48 hours, requirement for renal replacement therapy during 48 hours and hospital stay, time to extubation, and 30-day major morbidity endpoints including myocardial infarction, stroke, pneumonia, other infections, reoperation due to any reasons, length of ICU and hospital stay, and mortality were collected. Postoperative AKI which was diagnosed by the Acute Kidney Injury Network (AKIN) criteria (absolute increase in the serum creatinine concentration ≥0.3 mg/dL) within 48 hours of operation.^18^

### Study Endpoints

We investigated a possible optimal amount of urine output according to the association of urine output during CPB and the incidence of AKI. After separating cohort by the predicted optimal value, we performed logistic regression analyses in both sides to find potential predictors of AKI. And nomograms for estimating individual probability of AKI development were drawn and validated.

### Statistical Analysis

After Kolmogorov–Smirnov test, numerical data with normal distribution were presented as mean and standard deviation (SD). Patients who did and did not develop AKI were compared using the *t* test. Data that did not show normal distribution were expressed as median and interquartile range (IQR) and were compared using Mann–Whitney *U* test. Categorical variables were expressed as percentages and compared using Fisher exact test or chi-square test.

After calculation of the probability of the incidence of AKI according to CPB urine output, we constructed a cubic spline curve. Associations between risk factors and AKI incidence were evaluated using univariate logistic regression analysis repeatedly on entire patient group and subgroups with CPB urine output below and over the detected change point. For the multivariate model, a stepwise selection method was used to select significant variables when univariately significant (*P* = 0.05) factors were observed. The nomogram model was developed using the finally selected multivariate logistic model. The accuracy of the nomogram was internally and externally validated by discrimination and calibration, respectively. The discrimination of the model was measured using 1000 bootstrap samples to estimate bias-corrected concordance index, a measure of predictive accuracy of the model. The calibration of nomogram, which measures how far prediction is from observed outcome, was assessed with the calibration plot. Area under the curve (AUC) was constructed of the probability of the final model.

The statistical analyses were performed with SAS version 9.2 (SAS Institute Inc., Cary, NC) and R software version 2.15.3 using for creating the nomogram and receiver-operating characteristic (ROC) curve, and 2-side *P* value <0.05 was considered statistically significant.

## RESULTS

### Perioperative Data

Of 696 patients analyzed in the current study, AKI occurred in 257 patients (37%). Characteristics of the patients are listed in Table 1. Patients who developed postoperative AKI were older, shorter in height, had hypertension, diabetes, congestive heart failure, previous cerebral vascular accident, renal dysfunction, and undergone a previous cardiac surgery more frequently. Their additive EuroSCORE was significantly higher than that of patients who did not develop AKI. In addition, more patients in the AKI group had been taking rennin-angiotensin system blockers and digoxin before the surgery than those without AKI. With regard to operative features (Table 2), more patients with AKI underwent double or triple valve surgery, received inotropes and blood products, and had longer CPB and aortic cross-clamp time than those without AKI. The urine output during CPB was significantly lower, whereas the amount of hemofiltration was greater in patients with AKI. Diuretics were more frequently administered during surgery in patients with AKI. Postoperatively, urine output was lower during the first 24 hours in patients with AKI, but it was comparable during the 24 to 48 hours period between patients with and without AKI. Patients with AKI more often received blood transfusion during the first 48 hours and were administered greater amount of fluid during the 24 to 48 hours period. Myocardial infarction, stroke, reoperation, pneumonia, other infections, and mortality occurred more frequently in patients with AKI. The lengths of ICU and hospital stay were also significantly longer in patients with AKI. Additionally, the numbers of patients who required renal replacement therapy during the first 48 hours and in-hospital days were greater in patients with AKI (Table 3).

**TABLE 1:**
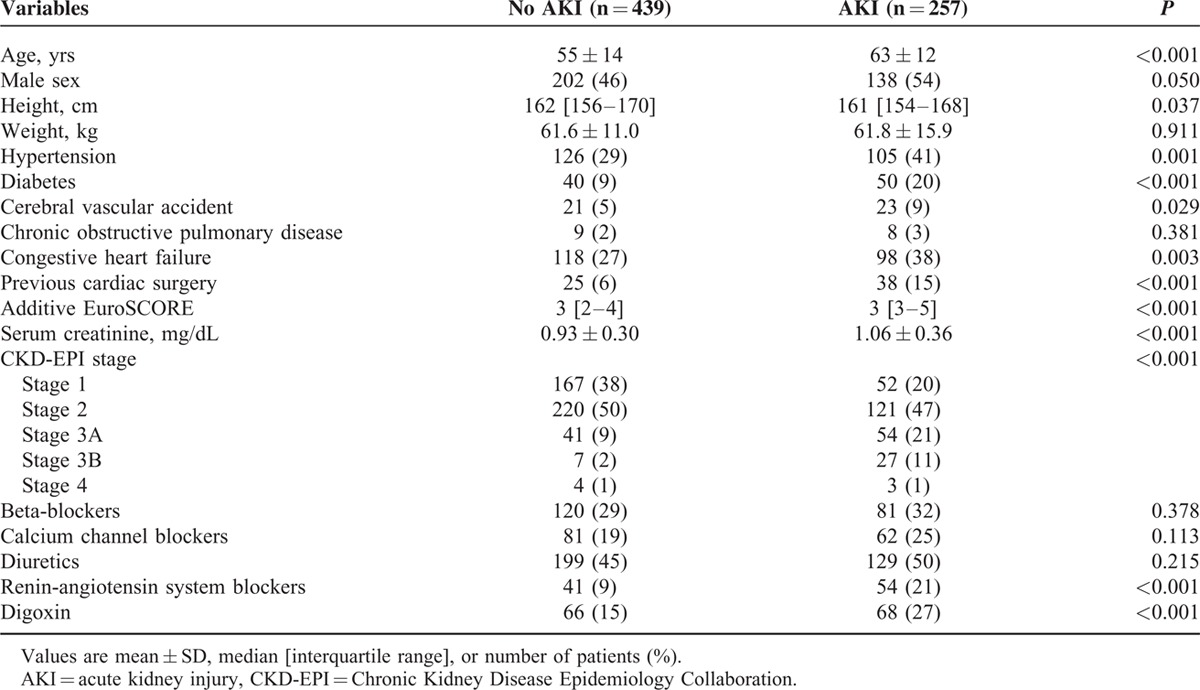
Characteristics of Study Populations

**TABLE 2:**
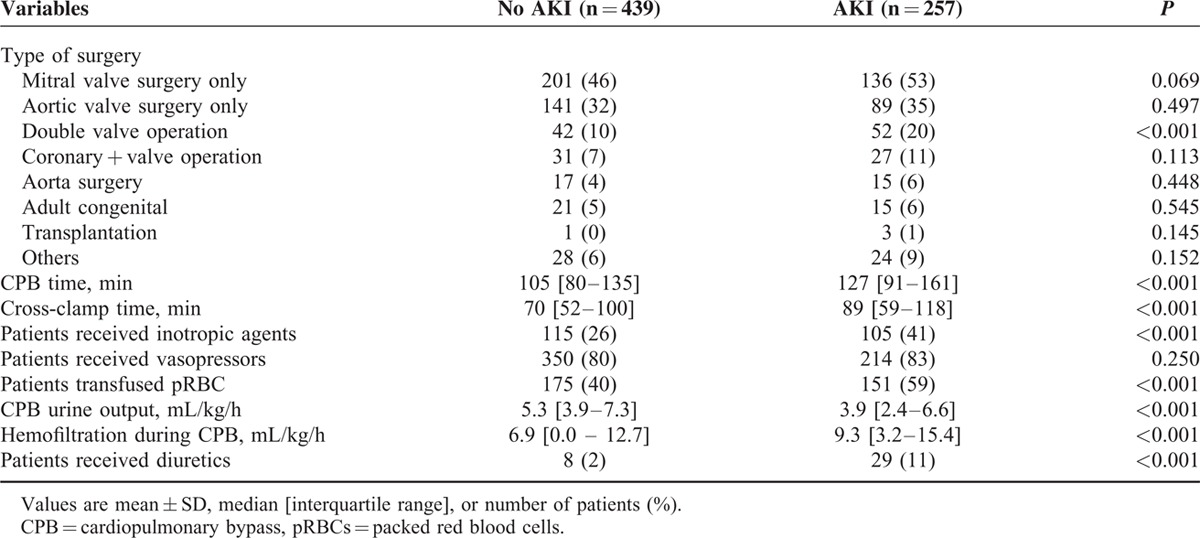
Operative Data

**TABLE 3:**
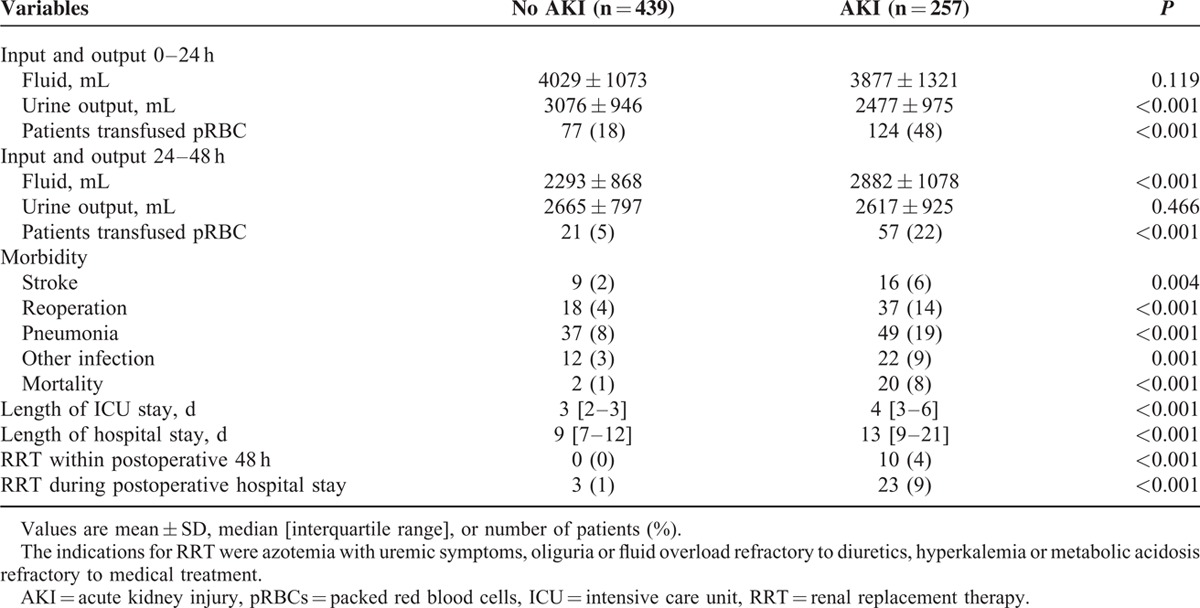
Postoperative Data

### Predictive Models for AKI

In a multivariate logistic regression analyses on all patients, male sex, lower preoperative eGFR, preoperative digoxin or diuretics use, double or triple valve surgery, intraoperative diuretics use, transfusion during the surgery, and number of transfused packed red blood cells (pRBCs) during surgery, number of transfused pRBCs during postoperative 48 hours were identified as statistically significant variables for predicting AKI (Table 4).

**TABLE 4:**
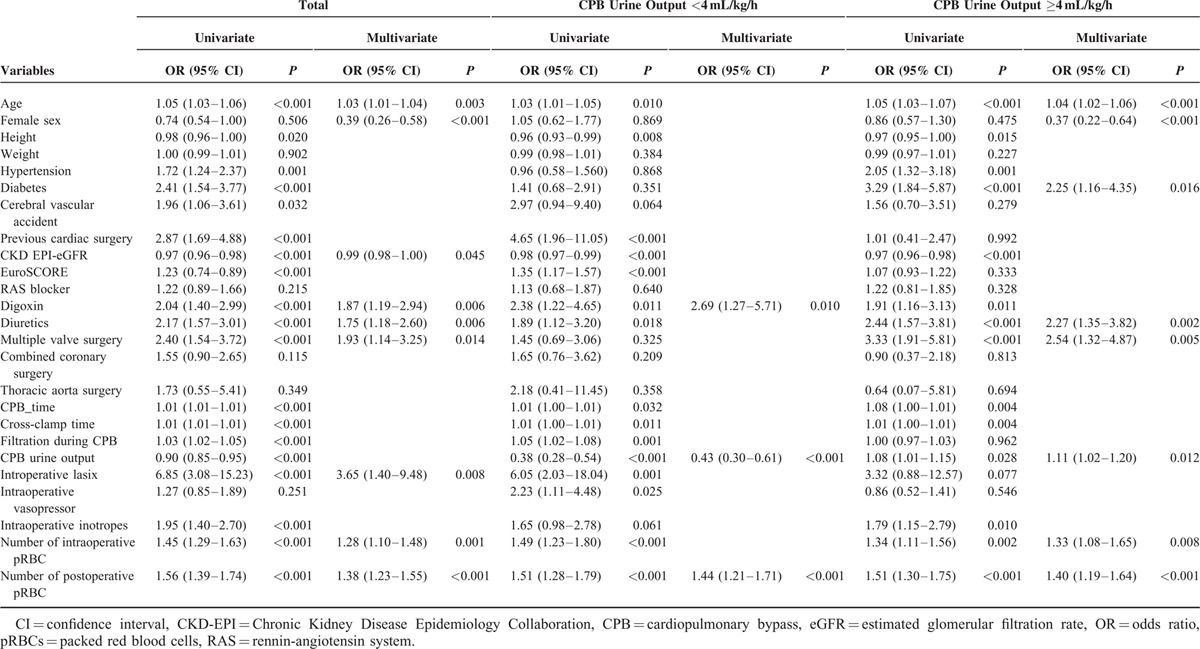
Predictive Power of Selective Variables for Development of Acute Kidney Injury

**FIGURE 1:**
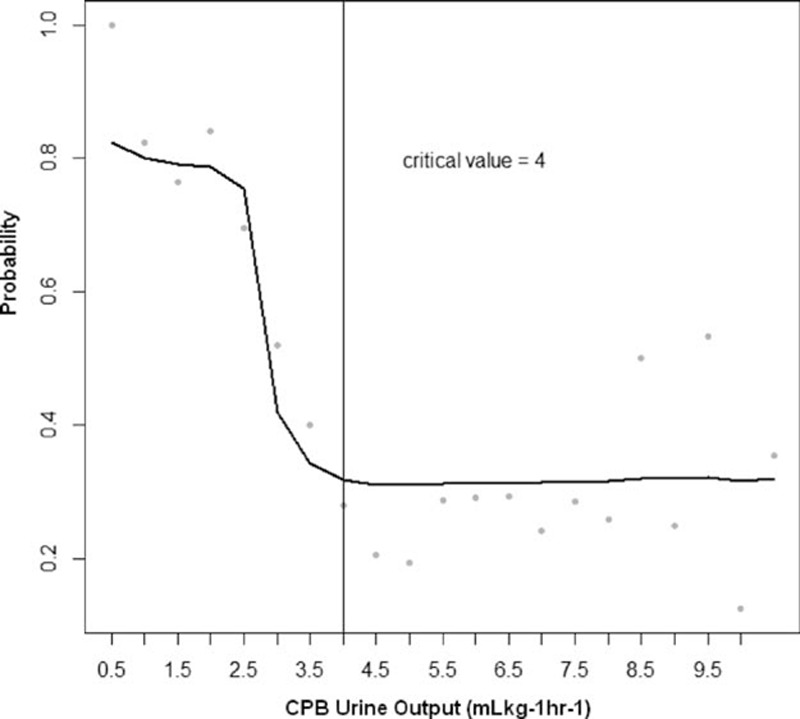
The change plot of acute kidney injury (AKI) development according to the urine output during cardiopulmonary bypass (CPB). The probability of AKI development continuously decreased when the amount of urine output during CPB was <4 mL/kg/h. The 4 mL/kg/h is a point at which the plot becomes flat.

In a cubic spline curve of the probability of AKI development according to the CPB urine output, 4 mL/kg/h was identified as a point at which the plot becomes flat (Figure 1). In the subgroup of 242 patients with CPB urine output <4 mL/kg/h, the logistic regression analyses identified, preoperative digoxin use, number of transfused pRBCs during postoperative 48 hours, and lower amount of CPB urine output as statistically significant variables. Logistic regression analysis on the subgroup of 453 patients with CPB urine output ≥4 mL/kg/h identified old age, male sex, diabetes, preoperative diuretics use, number of transfused pRBCs during surgery, number of transfused pRBCs during postoperative 48 hours, and higher amount of CPB urine output as statistically significant variables (Table 4). The odds ratio (OR) of the CPB urine output was 0.43 (95% confidence interval [CI] 0.30–0.61) in the former group and 1.11 (95% CI 1.02–1.20) in the latter group, respectively.

**FIGURE 2:**
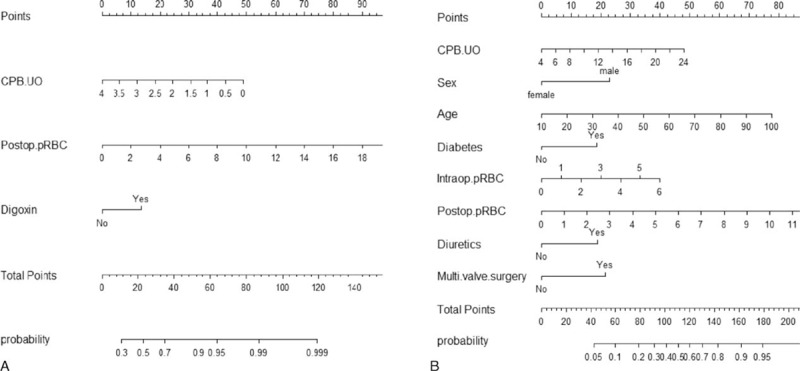
Nomograms for predicting the probability of acute kidney injury (AKI) development based on the fitted multiple logistic regression models in patients with urine output during cardiopulmonary bypass (CPB) <4 mL/kg/h (A) and ≥4 mL/kg/h (B). Directions for using the nomogram: The first row is the point assignment for each variable. For an individual patient, each variable is assigned a point value by drawing a vertical line between the exact variable value and the points line. Subsequently, a total point can be obtained by summing all of the assigned points for the variables. Finally, the predictive probability of AKI can be obtained by drawing a vertical line between “total points” and “probability” (the final row). CPB.UO = amount of urine output during CPB (mL/kg/^/^h), intraop.pRBC = number of transfused packed red blood cells during operation, multi.valve surgery = double or triple valve surgery, postop.pRBC = number of transfused packed red blood cells during postoperative 48 hours.

**FIGURE 3:**
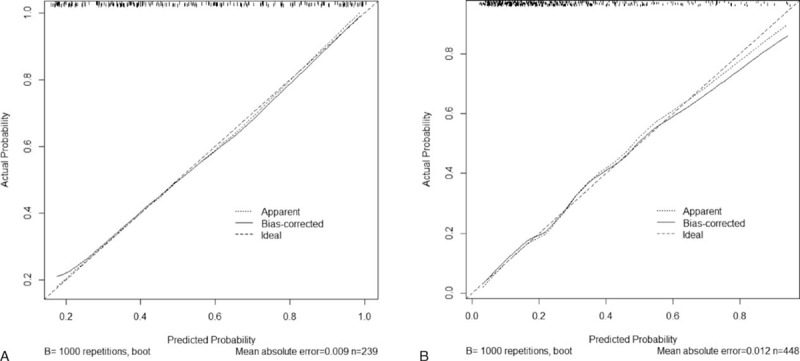
Calibration of the nomograms in patients with urine output during cardiopulmonary bypass (CPB) <4 mL/kg/h (A) and ≥4 mL/kg/h (B). The x-axis is the predicted probability from the nomogram, and the y-axis is the actual probability of acute kidney injury (AKI) development.

Figure 2 shows nomograms for predicting the probability of AKI development based on the logistic regression models in each subgroup. For a particular patient, points achieved for each predictive factor are summed to get the total points, which are used to locate the corresponding probability of AKI development from the bottom line. The calibration curves for the nomogram in the derivation set are shown on Figure 3, in which the x-axis is the predicted probability from the nomogram, and the y-axis is the actual proportion of AKI development.

**FIGURE 4:**
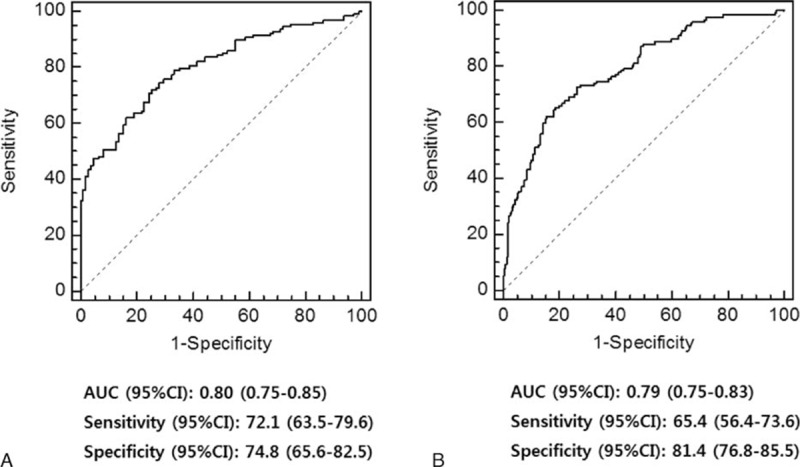
Receiver-operating characteristic curves based on the multivariate logistic regression models in patients with cardiopulmonary bypass (CPB) urine output <4 mL/kg/h (A) and ≥4 mL/kg/h (B). AUC = area under the curve, CI = confidence interval.

Figure 4 illustrates the ROC curves based on the multivariate logistic regression models in each subgroup. Area under the ROC curve of the predictive model in patients with CPB urine output <4 mL/kg/h was 0.80 (95% CI 0.75–0.85), with 72% sensitivity and 75% specificity (Figure 4A), and that in patients with CPB urine output ≥4 mL/kg/h was 0.79 (95% CI 0.75–0.83), with 65% of sensitivity and 81% of specificity (Figure 4B).

## DISCUSSION

In the present study, the amount of CPB urine output showed a biphasic association with the incidence of AKI. After dividing the patients by 4 mL/kg/h of the CPB urine output, predictive models for AKI showed excellent performance. To the best of our knowledge, this is the first study showing an independent association between the amount of CPB urine output and postoperative AKI.

Heart failure and low blood pressure, which are well-recognized predisposing factors of AKI after cardiac surgery, are closely associated with impaired renal perfusion during surgery.^19,20^ Renal hypoperfusion is the main factor precipitating acute renal failure after cardiac surgery,^21^ and urine flow is used as an indicator of renal perfusion during CPB and plays a role as guidance for the CPB management.^22^ Thus, poor urine output during CPB deserves to become an early indicator of renal injury in theory. However, none of the studies investigating the predictors of AKI has reported its significance,^12–14^ which seems to be because of the uniform analysis of the urine output. In addition to oliguria, the polyuria beyond a certain amount must be suspected to be abnormal because tubular damage during CPB can lead to the loss of urinary concentrating capacity.^23^

In the current study, we have plotted the probability of AKI occurrence in relation to the amount of CPB urine output and observed that there was a biphasic relationship between them. They were inversely proportional when the amount of CPB urine output was below 4 mL/kg/h. The probability remained constantly low and was not reduced further when the amount was over 4 mL/kg/h. Thus, we have defined it as a boundary value by which our cohort could be divided into groups to be analyzed separately. During CPB, hypothermia induces a decrease in tubular reabsorption of sodium and water.^24^ Reduced peritubular capillary oncotic pressure owing to extensive hemodilution also interferes with the active reabsorption of sodium and water.^25^ In addition, the use of mannitol as a priming solution also leads to the high urinary flow.^26^ Therefore, a diuresis is often developed during CPB even with a transient decline in renal function. Despite the completely different physiology, however, there has never been a comprehensive definition of oliguria during CPB. Several studies merely have mentioned 2 mL/kg/h as a threshold needing intervention,^27,28^ which was not based on the evidence or detailed calculation, but on the authors’ discretion. The adequate urine output associated with better postoperative renal prognosis should be obtained from their own database, because the absolute cut-off value may exclusively depend on the CPB and fluid management protocol of the institution. A point to be considered is that 3 to 4 mL/kg/h may be insufficient in the diuresis-prone CPB condition, as shown in our results.

We divided patients into 2 subgroups at 4 mL/kg/h of the CPB urine output. We could construct predictive and validated risk stratification models in both subgroups, and the amount of CPB urine output was identified as a predictor in both models. In the subgroup with CPB urine output below 4 mL/kg/h, the OR for AKI increased by 233% with every 1 mL/kg/h decrease in urine output. In patients with CPB urine output over 4 mL/kg/h, the OR for AKI increased by 11% with every 1 mL/kg/h increase in urine output. This finding underscores the need to carefully evaluate the decreased concentrating capacity resulting from the tubular damage, when faced with excessive diuresis.^29^ In the latter subgroup, preoperative use of diuretic agents was identified as another independent risk factor of AKI. Preoperative diuresis leading to the prerenal azotemia has been reported as a pathophysiologic factor in renal failure after cardiac surgery.^25^ Preoperative continuous diuretic therapy could have exaggerated diuresis during CPB and led to the consequent postoperative volume deficit deteriorating renal perfusion; however, their causal relationship is not clear. Diabetes was also identified as a risk factor in this subgroup, unlike in the other group. Among those with diabetic nephropathy, some might have preserved GFR preoperatively. This might be partly attributable to diabetes-induced hyperfiltration mediated by proximal tubular hyper-reabsorption (tubule glomerular feedback mechanism).^29^ Oxidative stress and inflammatory flare-up during CPB and cardiac surgery could have damaged tubular system and consequently unmasked the impaired glomerular filtration postoperatively. Preoperative digoxin use was closely associated with postoperative AKI regardless of the group. Most of the patients who had been taking digoxin preoperatively had suffered from atrial fibrillation in our study population, and the result is consistent with the previous study reporting that preoperative atrial fibrillation as a strong risk factor for postoperative renal dysfunction.^30^

Although there have been numbers of risk stratification models previously, those that do not incorporate weights of the risk factors may confront patients with excessive or insufficient intervention. In the current study, we additionally constructed nomograms to predict the probability of AKI development according to the individual risk based on the logistic regression models in each subgroup. The calibration curves for the nomograms (Figure 3) suggest excellent model calibration, with model estimates being close to observed rates. Our nomogram models may be helpful to determine patients at high risk of postoperative AKI in cardiac surgery who will benefit from early intervention.

Our study has some limitations. Firstly, as an inherent weakness of a retrospective study, we excluded patients for whom we could not obtain complete information from this analysis, which could possibly create selection bias. Secondly, our results do not guarantee the usefulness of CPB urine output to diagnose postoperative AKI in all cardiac surgical patients until it is validated with the database of diverse cardiac surgical cohorts.

In summary, the current study demonstrates primary evidence regarding the predictive relevance of CPB urine output to the risk of postoperative AKI. Assessment of the amount of urine output during CPB may be the most simple and fastest diagnostic method, but a comprehensive approach is required. External validation and further prospective studies relating to the biomarker assay are warranted to determine its clinical utility.
